# Virulence of Five Root-Knot Nematodes (*Meloidogyne* spp.) on Nine Industrial Hemp (*Cannabis sativa* L.) Varieties and Nematicidal Potential of Hemp Seed Extracts Against *Meloidogyne javanica*

**DOI:** 10.3390/plants14020227

**Published:** 2025-01-15

**Authors:** Dionysios Ntinokas, Ioannis Roussis, Antonios Mavroeidis, Panteleimon Stavropoulos, Antigolena Folina, Ioanna Kakabouki, Emmanuel A. Tzortzakakis, Dimitrios Bilalis, Ioannis O. Giannakou

**Affiliations:** 1 Laboratory of Agricultural Zoology and Entomology, Department of Science of Crop Production, Agricultural University of Athens, 11855 Athens, Greece; giannakou@aua.gr; 2 Laboratory of Agronomy, Department of Crop Science, Agricultural University of Athens, 11855 Athens, Greece; roussis@aua.gr (I.R.); antoniosmauroeidis@gmail.com (A.M.); stavropoulosp@aua.gr (P.S.); folina@aua.gr (A.F.); i.kakabouki@aua.gr (I.K.); 3 Institute of Olive Tree, Subtropical Crops and Viticulture, Department of Viticulture, Vegetable Crops, Floriculture and Plant Protection, ELGO-DIMITRA, 71307 Heraklion, Greece; tzortzakakis@elgo.iosv.gr

**Keywords:** *Cannabis sativa*, *Meloidogyne*, host–parasite relationships, hemp seed extracts, nematicidal activity, root-knot nematode control

## Abstract

The most common and damaging plant parasitic nematodes are root-knot nematodes (RNK). Although hemp has been clearly infected by RNK, little information is available regarding the extent of the damage and losses caused. In addition, no information is available concerning hemp seed extracts’ activity against RNK. In the current research, a greenhouse experiment was developed to evaluate the infection effect of five RKN species (*Meloidogyne javanica*, *M. incognita*, *M. arenaria*, *M. hapla*, and *M. luci*) in nine industrial hemp varieties (Fedora 17, Ferimon 12, Futura 75, Santhica 27, Santhica 70, KC Dora, KC Zuzana, Zenit, and USO 31), as was a laboratory experiment to evaluate the nematicidal activity of aqueous and water extracts of hemp seeds against second-stage juveniles (J2s) of *M. javanica*. The results revealed that the five evaluated *Meloidogyne* species were pathogenic to hemp plants. The lowest shoot weights were recorded in plants that were inoculated with *M. javanica* and *M. luci* (4.65 and 4.53 g, respectively). As for the root system, the most severe damage of the roots was detected in *M. incognita*-infected plants, presenting the lowest root weight (1.72 g). Regarding hemp varieties, the most susceptible were USO 31, Fedora 17, Ferimon 12, and Zenit, while Futura 75 hosted the lowest nematode numbers, including the lowest values of females per gram of root (10.66), number of egg masses per gram of root (4.70), and fecundity (33.68 eggs per egg mass). Moreover, this research showed that aqueous and ethanolic hemp seed extracts can control *M. javanica*. After 24, 48, 72, and 96 h of exposure to high concentrations (250–2000 ppm) of ethanolic and aqueous extracts, the J2s were permanently paralyzed; however, at low concentrations, only the ethanolic extract was effective. The ethanolic extract paralyzed J2s at concentrations higher than 62.5 ppm and reduced the egg hatching by up to 76% compared to the control treatment. In general, the efficacy of the aqueous extract was considerably lower than that of the ethanolic extract.

## 1. Introduction

Hemp (*Cannabis sativa* L., Cannabaceae family) is one of the oldest domesticated crops and has been cultivated for centuries, since it is adaptable to a variety of environments [1,2,3,4]. Several products are produced from its cultivation, including textile fibers, food, seed oils, and medicines [3,5,6]. In addition to producing high-quality textiles and seeds with a high nutraceutical value, hemp contains hundreds of secondary metabolites with medicinal properties [6,7,8]. Although hemp is versatile, legal restrictions have resulted in its prohibition due to the psychoactive secondary metabolite Δ^9^-tetrahydrocannabinol (THC) [3]. Nevertheless, there has been significant interest in the crop in recent decades as a result of its reauthorization [9,10,11]. Currently, hemp is grown in more than 40 countries around the globe, including several member states of the European Union [4,12]. Numerous new products are based on hemp, such as shampoos, toothpaste, facial and care products, plant-based proteins, tea, flowers, and many others [5,6]. As a result, the demand for the raw material has increased, and farmers are finding opportunities for a new profitable cultivation.

At this point, it is important to note that hemp cultivation is established in fields that were previously cultivated with crops that are susceptible to plant parasitic nematodes (PPNs). This means that many of these fields, which are going to be cultivated with hemp, are infested by PPNs. PPNs are regarded as one of the key limiting factors, accounting for around 10% of annual global crop yield losses [13]. Therefore, it is essential to investigate whether existing PPNs in various farms could cause problems in hemp cultivation. Until recently, research on hemp was limited due to the legal constraints mentioned above, and this area of research only started in the last few years [13,14]. Before the 1990s, and some time after, the international bibliography on hemp nematodes included primarily reports of their incidence or relationship, with little or no meaningful data on pathogenicity [14]. The increasing demand for medicinal cannabis during the last decade, as well as the increased cultivation of industrial and seed hemp, necessitates a contemporary approach to the possible impact of pests and diseases on crop health [15,16]. According to the available literature, some PPNs are reported to cause infections in hemp cultivation. Specifically, *Meloidogyne* (root-knot nematodes) and *Pratylenchus* (lesion nematodes) are the most frequently found genera on hemp, while *Ditylenchus* (stem nematode) has also been noted occasionally [13].

Root-knot nematodes (RKNs) are the most common and devastating PPN, with one or more species being found in almost every crop-growing region on the globe. RKN infections on hemp have been firmly confirmed, but the level of damage and losses has not been thoroughly studied [14]. Among the numerous reported RKN species, five have been investigated at least precipitately for their potential to reproduce on hemp: *Meloidogyne javanica*, *M. hapla*, *M. incognita, M. enterolobii*, and *M. chitwoodi* [13,14,17,18,19,20]. The first reports of *Meloidogyne javanica* and *M. enterolobii* infecting hemp in China were published in 2017 [17] and 2021 [18], respectively. As reported by Bernard et al. [14], in a comprehensive review, five *Meloidogyne* species have been found to be associated with hemp. These are the three cosmopolitans *M. hapla*, *M. javanica*, and *M. incognita* and two species, *M. enterolobii* and *M. chitwoodi*, with local interest. Recently, Desaeger et al. [19] reported *M. javanica* to be the dominant nematode species in central Florida, while Lawaju et al. [20] identified *M. incognita* Race 3 to be the causal agent for a hemp cultivation showing symptoms.

So far, the management of RKNs in infested fields has mainly been based on the use of synthetic nematicides. However, the number of active substances that are allowed for plant protection decreased under Directive 91/414/EEC (followed by Regulation 1107/2009/EC), resulting in the ban and withdrawal of many pesticides from the EU market due to environmental and health concerns [21]. Researchers are seeking new methods of nematode control and are trying to adopt integrated nematode management systems [22]. In the last decade, there has been an increased interest in the use of essential oils and/or botanical nematicides [23,24]. The lack of inherent resistance mechanisms in many crops, hemp included, and the high cost of preventative strategies make the control of nematodes difficult [14]. Thus, there is a need for alternative and safer methods for nematode control. Kayani et al. [25] reported that the incorporation in soil of pulverized leaves of *C. sativa* (hemp) caused a reduction in nematode infection and reproduction and improved the plant growth of cucumber. Second-stage juveniles (J2s) of pea cyst nematodes were killed when treated with aqueous leaf extracts of *C. sativa* [26].

In view of the above, the objectives of the present study were to determine (1) to what extent five RKN species can infect and multiply in nine hemp varieties, (2) the nematicidal activity of aqueous and water extracts of *C. sativa* seeds on second-stage juveniles (J2s) of *Meloidogyne javanica*, and (3) the differentiation activity of the extracts on egg hatching.

## 2. Results

### 2.1. Virulence of Five Meloidogyne Species on Nine Industrial Hemp Varieties

#### 2.1.1. Influence on Shoot Development

The shoot development of each variety used, as influenced by the specific root-knot species, is presented in Table 1. The shoot weight was significantly reduced in all RKN-inoculated plants compared with the control plants (*p* < 0.001). As for the nematode-inoculated plants, their plant growth was less affected when the hemp plants were inoculated with *M. arenaria* and *M. incognita* (5.38 and 5.36 g, respectively) compared to plants that were inoculated with *M. javanica* and *M. luci* (4.65 and 4.53 g, respectively). The plant growth of the plants that were inoculated with *M. hapla* was affected (with a mean value of 5.02 g) but to a lesser extent compared to those that were inoculated with the two previously mentioned species.

#### 2.1.2. Influence on Root Development

The highest root weight was recorded in untreated (control) plants (2.86 g). As for the RKN-inoculated plants, the highest root weight was presented in those that were inoculated with *M. arenaria* (2.06 g), which was significantly different only with those that were inoculated with *M. incognita*. This reduction in root weight was caused by inadequate lateral development as a result of the high infection rate caused by the different RKN species. In addition, significant differences were also revealed for the root development among the different varieties, with the highest values being found in the Futura 75 variety (2.29 g). Furthermore, as was also observed in the shoot development, a significant interaction was found among plant variety and nematode species (Table 2).

#### 2.1.3. The Development of Nematodes in Roots

Nematodes were found in all roots of the cultivars that were used, as presented in Table 3. There is strong evidence that less *M. hapla* nematodes were developed in cannabis plants than the other four species that we tested (Table 3). The highest number of nematodes was observed in plants that were inoculated with *M. javanica* (31.18 females per gram of root), followed by *M. luci* and *M. incognita* (20.42 and 20.40 females per gram of root, respectively). A relatively low number of females was recorded in hemp plants that were inoculated with *M. hapla*, presenting a mean value of 7.41 females per gram of root. A higher number of adult feeding females can cause significant damage by limiting the root system, which can lead to a reduced rate of nutrient and water uptake and therefore reduced plant growth development. This is also proven by the significant negative correlation of females per gram of root with the shoot and root weight (*r* = −0.4435, *p* < 0.001 and *r* = −0.5842, *p* < 0.001, respectively). As for plant varieties, the lowest number of nematodes was recorded in the Futura 75 cultivar (10.66 females per gram of root), while the highest one was found in the USO 31 cultivar (28.97 females per gram of root).

#### 2.1.4. Number of Egg Masses per Gram of Root

The results for the number of egg masses per gram of root in the different varieties of cannabis that we used are presented in Table 4. A significantly higher number of egg masses was produced by the species *M. javanica* (23.60), while the number of egg masses was considerably lower (reduced by 70%) in the species *M. incognita* and *M. luci* (9.93 and 8.82, respectively). Significantly lower numbers of egg masses were produced in plants that were infected with *M. arenaria* and *M. hapla* (4.04 and 3.29, respectively). Fedora 17, Ferimon 12, and Zenit supported higher root-knot populations, but the most susceptible, compared to all the other varieties, was USO 31 (Table 4). Contrast tests revealed that the lowest number of egg masses (0.13) was produced in KC Zuzana roots that were inoculated with *M. arenaria*, while the highest one (43.36) was recorded in USO 31 roots that were inoculated with *M. javanica*. In the present study, the regression analysis showed that females per gram of root and number of egg masses per gram of root presented a positive strong correlation (*r* = 0.7763, *p* < 0.001). Therefore, there was a good correlation among the severity of the RKN-induced disease (susceptibility) from the different *Meloidogyne* species and the rate at which RKNs reproduced in the evaluated hemp varieties.

#### 2.1.5. Influence on Nematode Fecundity

The fecundity (number of eggs per egg mass) of *M. javanica* females was significantly higher (74.11) in comparison with the other species (Table 5). Also, *M. incognita* and *M. luci* showed a high rate of reproduction (44.83 and 40.07, respectively), which was significantly different to those recorded for *M. hapla* and *M. arenaria* (30.72 and 22.54) (*p* < 0.0001). However, there was no a clear outcome in terms of the fecundity among the varieties tested, although significant differences between varieties were observed. Further statistical analysis using contrast tests showed that the lowest fecundity was recorded in the varieties KC Zuzana and Futura 75 when they were infected with *M. arenaria* (1.67 and 7.67, respectively).

### 2.2. Influence of Hemp Seed Extracts Against M. javanica

#### 2.2.1. The Effect of the Aqueous Extract on J2s Mobility

**Figure 1:**
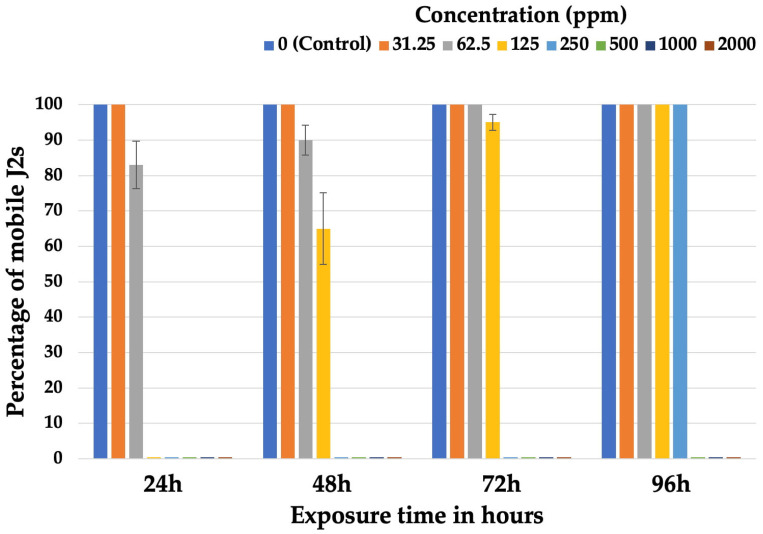
Percentage of mobile second-stage juveniles (J2s) after exposure for 24, 48, 72, and 96 h to different concentrations of aqueous extracts of *Cannabis sativa* seeds. Error bars represent standard errors of mean values.

The aqueous extract paralyzed 100% of the J2s at concentrations of 2000, 1000, 500, 250, and 125 ppm after 24 h of exposure (Figure 1). After 48 h of incubation, J2s exposed to a concentration of 125 ppm were mobile at a rate of 65%, which increased to 95% and 100% after incubation for 72 and 96 h, respectively. Juveniles that were exposed to a concentration of 62.5 ppm, while immobile at a rate of 83% after 24 h of incubation, showed an increase in the percentage of mobile juveniles in subsequent measurements (48 to 96 h of incubation), although this increase was not statistically significant (*p* > 0.05). In contrast to the observations at the aforementioned concentrations, when the J2s remained at a concentration of 250 ppm, they were immobile in all three observations (24 to 72 h of incubation), while 100% were mobile in the last measurement (96 h). The juveniles of the untreated control remained mobile throughout the experiment, whereas J2s that remained in concentrations of 2000, 1000, and 500 ppm were immobile throughout the duration of the experiment.

#### 2.2.2. The Effect of the Ethanolic Extract on J2s Mobility

**Figure 2:**
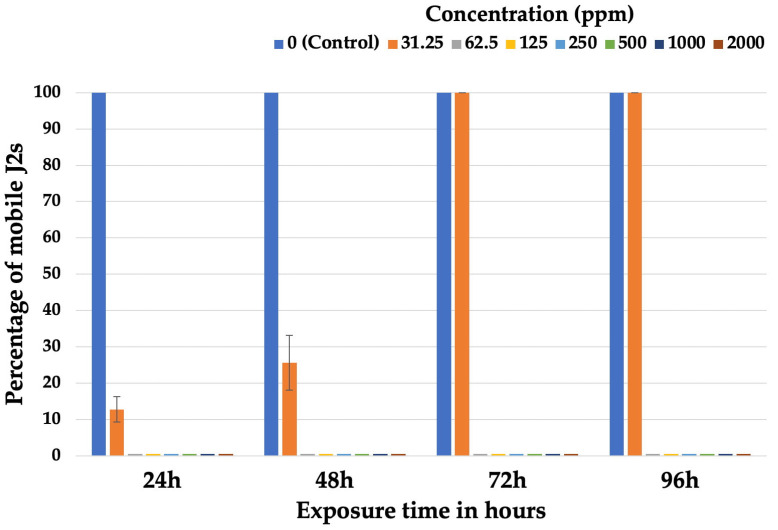
Percentage of mobile second-stage juveniles (J2s) after exposure for 24, 48, 72, and 96 h to different concentrations of ethanolic extracts of *Cannabis sativa* seeds. Error bars represent standard error of mean values.

Ethanolic extracts of cannabis seeds immobilized the nematodes to a large extent at all the tested concentrations from the first observation (24 h). In contrast to what was observed at certain concentrations of aqueous extracts, no reactivation of J2s was observed at any concentration of ethanolic extracts as the exposure time increased, as shown in Figure 2. The ethanolic extract proved to be more effective in immobilizing J2s compared to the aqueous extract. Mobile J2s were only recorded in the control and at the lowest tested concentration of 31.25 ppm. At this concentration, while a low percentage of mobile J2s was observed in the first two measurements, it subsequently increased, reaching 100% after 72 and 96 h. At higher concentrations (62.5–2000 ppm), all J2s were immobile from the first (24 h) to the last (96 h) measurement.

#### 2.2.3. Effect of Aqueous Extract on Egg Hatching

**Figure 3:**
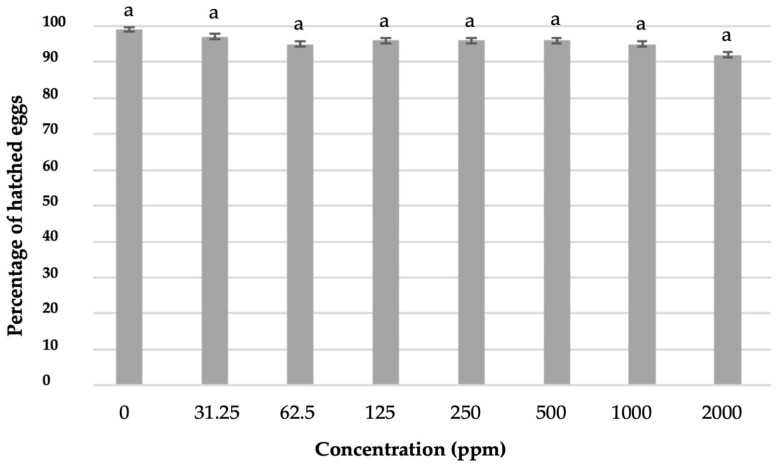
Percentage of hatched eggs after exposure of egg masses for seven days to different concentrations of aqueous extract solutions and transferal to clean water for another 21 days. Bars followed by different letters are significantly different according to Least Significant Difference (LSD) test (*p* ≤ 0.05). Error bars represent standard error of mean values.

As shown in Figure 3, the aqueous extract did not reduce the hatching of eggs (*p* > 0.05). No significant differences were recorded between the untreated control and the treatments or amongst the different concentrations of the aqueous extract. Similar results were observed between the highest concentration used (2000 ppm) and the lower concentrations, as well as with the control.

#### 2.2.4. Effect of Ethanolic Extract on Egg Hatching

**Figure 4:**
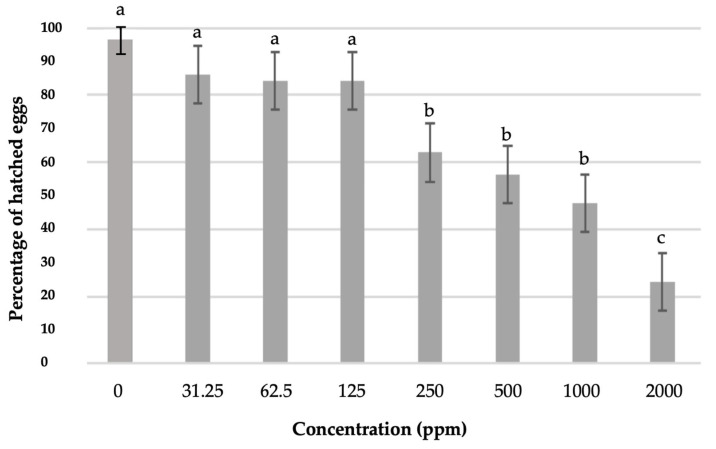
Percentage of hatched eggs after exposure of egg masses for seven days to different concentrations of ethanolic extract solution and transferal to clean water for another 21 days. Bars followed by different letters are significantly different according to Least Significant Difference (LSD) test (*p* ≤ 0.05). Error bars represent standard error of mean values.

Contrary to the aqueous extract, the ethanolic extract significantly reduced the hatching of eggs (Figure 4). The highest inhibition of egg hatching was observed at a concentration of 2000 ppm, followed by concentrations of 1000 ppm, 500 ppm, and 250 ppm, which also significantly reduced the hatching of J2s (*p* < 0.001). Specifically, the concentration of 2000 ppm reduced the hatching rate to 24%, while the immediately higher hatching rate was 47% at the concentration of 1000 ppm. Lower concentrations (125, 62.5, and 31.25 ppm) reduced egg hatching but not significantly and did not differ statistically significantly from each other; they did, however, differ from the control.

## 3. Discussion

Our study supports findings by other researchers showing that hemp is a good host for *M. incognita* [27,28,29], *M. javanica* [17,30,31,32], and *M. hapla* [28,33]. Based on the results as reported above, it can be concluded that the final root-knot population is dependent on the variety but also on the specific *Meloidogyne* species. However, the main outcome from our experiments is that the species of *Cannabis sativa* is a good host of root-knot nematodes. All tested cultivars were good hosts of *Meloidogyne* spp. based on the number of females that were observed in the roots. Our results are in agreement with those of Coburn and Desaeger [33], who reported that hemp is a good host for certain RKN species in the USA, and this can be a limiting factor for hemp production in Florida. Also, our findings are in agreement with other researchers from the USA and China showing that hemp can support substantial populations of the cosmopolitan *M. incognita*, *M. hapla*, and other species of local importance such as *M. enterolobii* [27,28,29,33,34]. This could create a practical problem, since many new plantations are established in farms that were previously cultivated with crops that are susceptible to any of these species parasitizing hemp plants.

Although the experiment was conducted in pots, which means a very limited space, the canopy development was affected by the presence of root-knot species at different levels. This means that in field conditions, this effect could be more severe and affect the hemp production to a higher degree. It seems that the canopy development is correlated with that of the roots, since in *M. arenaria* treatments, higher plant development was coupled with a larger root system. However, as stated by Coburn and Deseager [33], differences in plant development between cultivars are due to the physiology of the plants.

In addition, it seems that *Cannabis sativa* is more susceptible to *M. javanica* than the other tested RKN species. This might be a constraint, since this RKN species is cosmopolitan and one of the most common in Greek farms [24,35]. On the other hand, the lowest number of nematodes per gram of root was recorded in *M. hapla*-infested plants. However, this could be unrelated to the specific cultivars that were used in the present trials but rather to the *M. hapla* biology. *Melodogyne hapla* is a more psychrophilic species compared to the others. The temperature of the greenhouse (25–29 °C) might not be favorable for *M. hapla* development, which was reflected in the number of nematodes that had developed by the end of the life cycle. The greenhouse temperature, based on the work published by Tyler [36], was close to the optimum for the thermophilic species such as *M. javanica* and *M. incognita*. Hence, no straight comparison as far as root and canopy development of different cultivars is concerned can be carried out. Coburn and Deseager [33] reported that the host-specific origin of the RKN isolate populations might affect the life cycle in hemp plants. A *M. hapla* that they worked with was not virulent to hemp, since it was isolated from strawberry cultivation. However, this hypothesis should be tested to reach a definite conclusion. Our populations (*M. javanica/incognita*) originated from tomato (*Lycopersicon esculentum* Mill.) plants, while *M. arenaria* was originally isolated from balm (*Melissa officinalis* L.) and *M. hapla* from lavender (*Lavandula angustifolia* L.). However, all species were maintained on tomato plants for many years. *M. luci* was originally isolated from kiwi plants. Whether or not this fact could affect nematode virulence in hemp plants must be further tested.

As hemp becomes a reliable, large-acreage crop with a high return on investment, there are many useful avenues for research to explore. This plant species produces a variety of unique, biologically active molecules that may be screened for their effect on plant pathogens and particularly on nematodes [37]. According to previous studies, the extracts of inflorescences, leaves, and roots of hemp contain a wealth of secondary metabolites that are bioactive and have potential as sources of biopesticides; however, there is no information about the use of seed extracts for nematode management [33,37].

In the present study, the effect of the aqueous solution of hemp seeds against *M. javanica* was evident from a concentration of 125 ppm after 24 h of incubation. However, it seems that the effect was reversible, because the percentage of mobile juveniles increased in the measurement after 48 h of incubation, while the percentage of mobile juveniles further increased after incubation for 72 and 96 h. The same occurred at a concentration of 250 ppm, which immobilized the juveniles after incubation for up to 72 h, but surprisingly, all the juveniles were mobile during the measurement after 96 h of incubation. The hypothesis that can be made is that the aqueous solution from cannabis seeds possibly contains volatile components that do not affect nematodes at low concentrations. In contrast to the aqueous solution, the ethanolic solution was very effective in terms of juvenile movement. The same phenomenon as with the aqueous solution, regarding the reversal of its effect, occurred with the ethanolic solution of hemp seeds, but to a lesser extent and only for the low concentration of 31.25 ppm. All other tested concentrations were very effective against the juveniles, and their effect was maintained until the end of the experiment after 96 h of incubation.

The incubation of the egg masses in the aqueous solution did not affect the eggs’ ability to hatch at all. Similar hatching rates were observed among the different concentrations, which showed no difference either between them or when compared to the control. The effect of the ethanolic solution was entirely different compared to that of the aqueous solution. Specifically, the results clearly showed that it is a concentration-dependent phenomenon, with an increase in concentration from 125 ppm to 2000 ppm. At low concentrations (up to 125 ppm), there were no significant differences compared to the control. The effect of a substance in inhibiting egg hatching is significant, because a large portion of the nematode population is in this stage (eggs that are either free in the soil or inside the egg mass) at the beginning of the growing season when nematicide applications are made.

The nematicidal mechanism of hemp plants extracts or their components remains unclear. In the current research, cannabinoids may be responsible for the nematicidal properties of hemp. These cannabinoids are produced specifically by cannabis, which contains 21 carbon atoms in terpenophenolic compounds [38]. A total of over 60 types of cannabinoids have been identified, including cannabidiol, cannabinol, delta 9-tetrahydrocannbinol, tetrahydrocannbivarin, tetrahydrocannbinolic acid, etc. [39]. There is evidence that cannabinoids have antibacterial and antifungal properties [40,41], but their action against nematodes has yet to be proven [25,33]. Despite previous research conducted by McPartland and Glass [42], which demonstrated that cannabinoid receptors are not involved in the nematicidal effects of hemp, several other studies have been conducted on *Caenorhabditis elegans* in terms of alterations in behavior and physiology [32,33]. As a result of cannabinoid treatment, juveniles displayed a “dazed and confused” behavior, accompanied by delayed responses to aversive stimuli, decreased feeding rates, slowed locomotion, and increased unproductive turning [43]. According to this study, behavioral changes were modulated by complex signaling systems. There is also evidence that cannabinoids can have an effect on nematodes’ lifespans and health spans. A previous research work investigated the influence of cannabidiol on two strains of *Caenorhabditis elegans*, N2 and a transgenic line that was used to study Alzheimer’s disease (BZ555); this line expresses the amyloid-β (Aβ) protein throughout the nervous system [44]. The transgenic strain exhibits increased exploratory behavior in juveniles, increased pharyngeal pumping in aged animals, and an improved chemotaxis response, whereas wild-type animals exhibit only exploratory behavior [32,44]. Additionally, it is possible that seed extracts disrupt the cell membranes of nematodes and adversely affect their permeability. There are serious indications that the toxic chemicals in hemp may cause immobilization, mortality, poor penetration, and later retardation in different functions of second-stage juveniles, including feeding and reproduction [25]. To the best of our knowledge, this the first report of using seed extracts of hemp against RKNs and also the first report of the virulence of *M. luci* on hemp.

## 4. Materials and Methods

### 4.1. Nematode Cultures

All *Meloidogyne* species used in the present study were maintained in a greenhouse at 25–30 °C, with a 16 h photoperiod at Crete, Greece, for many years. These populations were multiplied using tomato plants (*Lycopersicon esculentum* Mill. cv. Belladona) at the five-leaf stage in a growth chamber at 25 ± 2 °C, with 65% RH and a 16 h photoperiod in 18 cm plastic pots containing a mixture of peat and perlite at the Agricultural University of Athens, Greece. The plants were maintained in these conditions for 50 days when uprooted, and the roots were carefully washed to remove soil particles. Eggs were extracted using the hypochlorite method [45] with a 1% sodium hypochlorite solution and were used for juvenile hatching. All second-stage juveniles (J2s) that were collected in the first three days were discarded, and then, fresh juveniles were collected every two days and used in the trials.

### 4.2. Hemp Plants

Industrial hemp plants (*Cannabis sativa* L.) were maintained in a plastic greenhouse at 25–29 °C, 65% RH, and a 16 h photoperiod at the Agricultural University of Athens, Greece (latitude 37°59′ N, longitude 23°42′ E, 30 m from the sea surface). The plants were seeded in mid-April 2021 and grown in 9.5 L plastic pots (24 cm diameter) filled with a mixture of compost and perlite at a ratio of 5:1 (*v*/*v*). After 30 days in these conditions, the plants were inoculated with a suspension of J2s. All plants were maintained for another 30 days in the plastic greenhouse and then uprooted and washed free of soil.

### 4.3. Virulence of Five Meloidogyne Species on Nine Industrial Hemp Varieties

To monitor the development of root-knot nematodes on industrial hemp plants, *Meloidogyne incognita*, *M. javanica*, *M. arenaria*, *M. hapla*, and *M. luci* were used to inoculate nine different varieties of hemp (Table 6) according to a two-factor completely randomized design. The cannabis varieties that were used in the present study were selected based on the fact that they are the most popular cultivated varieties in the European Union and suitable for Greek cultivation conditions, where this experiment was set up. In general, a total of forty-five treatments were carried out, with five pots being planted in each treatment. The hemp plants were prepared as described in the previous paragraph and inoculated with a 5 mL suspension containing 500 J2s. The suspension was delivered with a pipette in three 2 cm holes around the plant stems. The soil in the pots was covered with aluminum foil for two days to avoid water evaporation and provide the appropriate time for the J2s to enter the roots. After that period, each pot was irrigated with 200 mL of tap water at a two-day interval. No fertilizers and pesticides were used throughout the cultivating period. After 30 days, the plants were uprooted and carefully washed free of soil. The aboveground part was separated from the root, and both parts were weighed after removing the excess of moisture from the roots. All roots were stained using acid fuchsin [46]. After that, the roots were washed to remove the excess of stain using tap water and placed in vials with a solution of glycerol and distilled water at a ratio of 1:1 (*v*/*v*). Just before counting, roots were cut into small pieces of 1–2 cm, and all female nematodes inside the roots and egg masses were counted in the whole root system using a stereoscopic microscope (Leica Wild M3B, Heerbrugg, Switzerland) at 12.5× magnification. Ten egg masses were removed and placed in 10 mL hypochlorite solution (10% in NaCl) and left for 3–4 min. Then, the solution was vortexed and rinsed with tap water to remove hypochlorite using a 38 μm sieve. All eggs from the sieve were collected in 10 mL vials and counted under a stereoscopic microscope at 200×.

### 4.4. Preparation of Extracts

The hemp seeds were grinded by using a mortar and pestle, and the material was maintained in a fridge at 4 °C for one week. Aqueous and ethanolic solutions of hemp seeds were tested for J2 motility at concentrations of 62.5, 125, 250, 500, 1000, and 2000 ppm. An aqueous solution of hemp was prepared by mixing 25 g of the above material in 250 mL of distilled water. It was homogenized and placed in a beaker and left at room temperature for 24 h. The above suspension was centrifuged at 9000 rpm for 10 min to separate the solid phase, and the liquid supernatant was removed and used for the experiments. The ethanolic solution was obtained by mixing 4.5 g of the material in 45 mL of absolute ethanol (≥99.5% *v*/*v*; EMPARTA^®^ ACS reagent, for extraction and analytical purposes). It was homogenized using a mortar and pestle and left in a beaker for 2 h at room temperature. The suspension was poured through a filter paper (Ø 110 mm, 2–3 μm), and the resulting solution was placed in a glass beaker. A stock solution at a concentration of 4000 ppm was prepared, and then, different solutions were prepared by gradually diluting a certain volume of the stock solution with distilled water containing polyoxyethylene (20) sorbitan monolaurate (Tween-20) (0.3%). All solutions were prepared at concentrations twice as high as the one tested.

#### 4.4.1. Motility Tests Using J2s

Subsequently, 1 mL of stock solution was mixed with 1 mL of nematode solution containing at least 50 J2s. Distilled water containing only J2s was served as the control. The plates (Cellstar^®^ 24-wells) were covered with lids to avoid water evaporation. All measurements of juvenile motility were conducted with the aid of an inverted microscope (Zeiss; Oberkochen, Germany) at 100× magnification after 24, 48, 72, and 96 h. The juveniles were categorized as mobile or paralyzed. Eight replicates were performed for each concentration, and the experiment was repeated twice.

#### 4.4.2. Hatching Tests Using Egg Masses

Hatching tests were conducted using mature egg masses, which contained a juvenile inside them and were collected from roots that were free of soil. Each egg mass was placed in handmade plastic extraction trays made from a 6 cm Petri dish. Aqueous and ethanolic hemp extracts at the concentrations of 62.5, 125, 250, 500, 1000, and 2000 ppm were used for the purpose of this study and prepared according to the aforementioned procedure. The single egg mass was maintained for seven days in the Petri dish, the solution was transferred to a beaker, and all hatched J2s were counted with the aid of a stereoscopic microscope (Zeiss, Oberkochen, Germany). Then, each extracting tray was filled with clean distilled water, covered with aluminum foil to avoid water evaporation, and placed in an incubator at 26 ± 1 °C. Hatched J2s were counted every seven days and discarded, and fresh water was added. The experiment was terminated after 4 weeks, since no J2s were emerging any longer. Each egg mass was squashed separately in a drop of water on a glass microscope slide, and the number of unhatched eggs per egg mass was counted using an inverted microscope (Zeiss, Oberkochen, Germany). The experiment was conducted twice, and each treatment was replicated five times.

### 4.5. Statistical Analysis

Data were subjected to Analysis of Variance (ANOVA) using the GLM test in SAS (SAS University Edition; SAS Institute Inc., Cary, NC, USA). Before statistical analysis, all numbers were calculated according to the Abbot formula:

(1) $$\alpha =\frac{mortality\;treatment-mortality\;water}{100-mortality\;water}\times 100\%$$

Treatment means were compared using the Least Significant Difference (LSD) test. All experiments were conducted twice, and data were combined and analyzed together, since no variation was revealed between data.

## 5. Conclusions

In conclusion, the present research work revealed that the most severe damage to the roots was detected in *M. javanica*-infected plants. As for hemp varieties, the most susceptible were Zenit, Santhica 70, and USO 31, while Futura 75 hosted the lowest nematode numbers. In addition, this research showed that hemp seed extracts can control *M. javanica*. Overall, the efficacy of the aqueous extract was considerably lower than that of the ethanolic extract; however, further studies are necessary to determine the most efficient rate and dose of these extracts.

## Figures and Tables

**Table 1:** Shoot weight of *Cannabis sativa* after inoculation with 500 s-stage juveniles (J2s) of five *Meloidogyne species* and 30 days of maintenance in greenhouse at 25–29 °C.

Plant Variety	Nematode Species						
	*M. javanica*	*M. incognita*	*M. arenaria*	*M. hapla*	*M. luci*	Control	Mean
	Shoot Weight (g)						
Fedora 17	3.71	5.44	4.19	3.43	2.94	5.86	4.26 d
Ferimon 12	4.07	4.99	4.89	4.26	4.38	5.90	4.75 cd
Futura 75	4.83	6.29	6.74	3.73	5.92	7.48	5.83 a
Santhica 27	4.68	6.50	5.08	5.75	4.78	6.92	5.62 ab
Santhica 70	5.91	7.46	5.06	4.34	4.70	7.67	5.86 a
KC Dora	5.67	4.88	5.59	5.98	4.58	6.65	5.56 ab
KC Zuzana	3.49	4.22	6.59	5.95	3.05	7.14	5.07 bc
Zenit	5.07	4.27	4.04	5.21	3.86	5.81	4.71 cd
USO 31	4.42	4.19	6.24	6.54	6.57	6.73	5.78 a
Mean	4.65 CD	5.36 B	5.38 B	5.02 BC	4.53 D	6.68 A	
**Source of Variation**							
Source			df	F-Ratio	*p*-Value	LSD_5%_	
Plant Variety			8	7.84	0.0001 ***	0.299	
Nematode Species			5	19.82	0.0001 ***	0.244	
Plant Variety × Nematode Species			40	3.04	0.0001 ***	0.732	

F-test ratios are from Analysis of Variance. ***: significant at the 0.1% probability level. The capital letters compare the mean values of nematode species, and the lowercase letters compare the mean values of plant varieties by the Least Significant Difference (LSD) test (*p* ≤ 0.05).

**Table 2:** Root weight of *Cannabis sativa* after inoculation with 500 s-stage juveniles (J2s) of five *Meloidogyne species* and 30 days of maintenance in greenhouse at 25–29 °C.

Plant Variety	Nematode Species						
	*M. javanica*	*M. incognita*	*M. arenaria*	*M. hapla*	*M. luci*	Control	Mean
	Root Weight (g)						
Fedora 17	1.39	2.00	1.25	0.95	1.41	2.20	1.53 c
Ferimon 12	2.19	1.54	1.83	1.42	2.12	2.61	1.95 b
Futura 75	1.67	2.18	2.65	1.50	2.47	3.29	2.29 a
Santhica 27	1.76	2.51	1.74	1.78	2.11	2.99	2.15 ab
Santhica 70	2.40	2.59	2.13	1.69	1.37	2.88	2.18 ab
KC Dora	2.03	1.47	2.74	2.78	1.45	2.94	2.24 ab
KC Zuzana	1.40	1.08	2.73	1.92	1.45	2.83	1.90 b
Zenit	2.47	1.19	1.54	2.21	1.47	2.74	1.94 b
USO 31	1.22	0.93	1.97	2.07	2.94	3.27	2.07 ab
Mean	1.84 BC	1.72 C	2.06 B	1.81 BC	1.87 BC	2.86 A	
**Source of Variation**							
Source			df	F Ratio	*p*-Value	LSD_5%_	
Plant Variety			8	3.58	0.0005 ***	0.172	
Nematode Species			5	18.24	0.0001 ***	0.140	
Plant Variety × Nematode Species			40	2.88	0.0001 ***	0.421	

F-test ratios are from Analysis of Variance. ***: significant at the 0.1% probability level. The capital letters compare the mean values of nematode species, and the lowercase letters compare the mean values of plant varieties by the Least Significant Difference (LSD) test (*p* ≤ 0.05).

**Table 3:** Females per gram of root of *Cannabis sativa* varieties after inoculation with 500 s-stage juveniles (J2s) of five *Meloidogyne species* and 30 days of maintenance in greenhouse at 25–29 °C.

Plant Variety	Nematode Species					
	*M. javanica*	*M. incognita*	*M. arenaria*	*M. hapla*	*M. luci*	Mean
	Females Per Gram of Root					
Fedora 17	46.82	23.02	23.61	12.90	26.07	26.48 ab
Ferimon 12	32.97	22.75	17.17	8.90	26.50	21.66 bcd
Futura 75	16.62	10.71	11.20	6.74	8.03	10.66 fg
Santhica 27	26.20	16.85	10.31	9.99	24.17	17.50 cde
Santhica 70	21.87	24.57	23.75	14.63	34.02	23.77 abc
KC Dora	20.82	15.24	10.00	2.78	18.23	13.41 ef
KC Zuzana	25.39	25.07	4.88	7.07	26.57	17.79 cde
Zenit	33.34	29.46	13.45	2.21	16.49	18.99 cde
USO 31	53.84	35.81	22.36	7.01	25.83	28.97 a
Mean	30.87 A	22.60 B	15.19 C	8.02 D	20.42 B	
**Source of Variation**						
Source		df	F-Ratio	*p*-Value	LSD_5%_	
Plant Variety		8	11.29	0.0001 ***	5.221	
Nematode Species		4	40.61	0.0001 ***	5.046	
Plant Variety × Nematode Species		32	1.84	0.002 **	11.233	

F-test ratios are from Analysis of Variance. ** and ***: significant at the 1% and 0.1% probability levels, respectively. The capital letters compare the mean values of nematode species, and the lowercase letters compare the mean values of plant varieties by the Least Significant Difference (LSD) test (*p* ≤ 0.05).

**Table 4:** Egg masses per g of *Cannabis sativa* root varieties after inoculation with 500 s-stage juveniles (J2s) of five *Meloidogyne species* and 30 days of maintenance in greenhouse at 25–29 °C.

Plant Variety	Nematode Species					
	*M. javanica*	*M. incognita*	*M. arenaria*	*M. hapla*	*M. luci*	Mean
	Number of Egg Masses per Gram of Root					
Fedora 17	36.69	9.95	4.15	4.15	11.02	13.19 b
Ferimon 12	24.28	11.26	7.10	2.33	9.80	10.95 bcd
Futura 75	13.44	5.73	1.04	1.71	1.58	4.70 fg
Santhica 27	18.54	6.68	2.20	3.88	11.93	8.64 cde
Santhica 70	17.68	13.55	5.82	4.21	18.60	11.97 bc
KC Dora	14.70	6.16	2.88	3.55	5.55	6.57 efg
KC Zuzana	18.44	11.75	0.13	2.25	7.34	7.98 def
Zenit	25.71	14.07	10.00	8.38	6.85	13.00 b
USO 31	43.36	19.04	5.82	2.60	11.33	16.43 a
Mean	23.65 A	10.91 B	4.34 C	3.67 C	9.33 B	
**Source of Variation**						
Source		df	F-Ratio	*p*-Value	LSD_5%_	
Plant Variety		8	9.72	0.0001 ***	2.343	
Nematode Species		4	90.82	0.0001 ***	4.647	
Plant Variety × Nematode Species		32	2.66	0.0001 ***	5.136	

F-test ratios are from Analysis of Variance. ***: significant at the 0.1% probability level. The capital letters compare the mean values of nematode species, and the lowercase letters compare the mean values of plant varieties by the Least Significant Difference (LSD) test (*p* ≤ 0.05).

**Table 5:** Fecundity of five *Meloidogyne species* on nine *Cannabis sativa* varieties after inoculation with 500 s-stage juveniles (J2s) and 30 days of maintenance in greenhouse at 25–29 °C.

Plant Variety	Nematode Species					
	*M. javanica*	*M. incognita*	*M. arenaria*	*M. hapla*	*M. luci*	Mean
	Fecundity (Number of Eggs per Egg Mass)					
Fedora 17	80.56	43.79	18.02	31.24	42.01	43.12 abc
Ferimon 12	74.08	48.08	47.00	17.57	36.80	44.71 abc
Futura 75	71.43	44.34	7.67	26.30	18.66	33.68 d
Santhica 27	67.18	46.24	13.03	29.44	49.09	41.00 bcd
Santhica 70	80.60	54.18	25.18	28.96	54.57	48.70 ab
KC Dora	74.35	41.21	24.98	24.25	31.24	39.21 cd
KC Zuzana	69.64	40.94	1.67	29.39	42.79	36.89 cd
Zenit	76.13	41.13	20.99	42.59	41.97	44.56 abc
USO 31	72.99	43.56	44.29	46.74	43.52	50.22 a
Mean	74.11 A	44.83 B	22.54 D	30.72 C	40.07 B	
**Source of Variation**						
Source		df	F-Ratio	*p*-Value	LSD_5%_	
Plant Variety		8	8.35	0.0001 ***	7.234	
Nematode Species		4	107.95	0.0001 ***	8.163	
Plant Variety × Nematode Species		32	2.09	0.0002 ***	9.732	

F-test ratios are from Analysis of Variance. ***: significant at the 0.1% probability level. The capital letters compare the mean values of nematode species, and the lowercase letters compare the mean values of plant varieties by the Least Significant Difference (LSD) test (*p* ≤ 0.05).

**Table 6:** Origin, sexual type, and vegetative cycle of the evaluated hemp varieties (cultivars) used in the current research (seed presented a legal THC content, i.e., <0.2 *w*/*v*).

Cultivar	Origin	Sexual Type	Vegetative Cycle
Fedora 17	France	Monoecious	Early (<125 days)
Ferimon 12	France	Monoecious	Early (<125 days)
Futura 75	France	Monoecious	Late (<145 days)
Santhica 27	France	Monoecious	Medium (<135 days)
Santhica 70	France	Monoecious	Late (<145 days)
KC Dora	Hungary	Monoecious	Late (<145 days)
KC Zuzana	Hungary	Monoecious	Early (<125 days)
Zenit	Romania	Monoecious	Early (<125 days)
USO 31	Ukraine	Monoecious	Early (<125 days)

Vegetative cycle: very early (<110 days), early (<125 days), medium (<135 day), late (<145 days), and very late (<160 days).

## Data Availability

Data is contained within the article.
